# Adding a Yeast Blend to the Diet of Holstein Females Minimizes the Negative Impacts of Ingesting Feed Naturally Contaminated with Aflatoxin B1

**DOI:** 10.3390/ani16020219

**Published:** 2026-01-12

**Authors:** Mario Augusto Torteli, Andrei Lucas Rebelatto Brunetto, Emeline P. Mello, Guilherme Luiz Deolindo, Luisa Nora, Tainara Letícia dos Santos, Luiz Eduardo Lobo e Silva, Roger Wagner, Aleksandro Schafer da Silva

**Affiliations:** 1 Graduate Program of Animal Science, Universidade do Estado de Santa Catarina (UDESC), Chapecó 89815-630, SC, Brazil; marioaugustovet@gmail.com (M.A.T.); andreibrunetto03@gmail.com (A.L.R.B.); emeline@unochapeco.edu.br (E.P.M.); tainaraleticia915@gmail.com (T.L.d.S.); 2 Graduate Program of Biochemistry and Molecular Biology, Universidade do Estado de Santa Catarina (UDESC), Lages 88520-000, SC, Brazil; guilhermeluizd@outlook.com (G.L.D.); luisa.nora22@gmail.com (L.N.); 3 Department of Food Science, Universidade Federal de Santa Maria, Santa Maria 97105-900, RS, Brazil; eduardo.lobo@acad.ufsm.br (L.E.L.e.S.); rogerwag@gmail.com (R.W.)

**Keywords:** mycotoxin, yeasts, prebiotic, animal health, weight gain

## Abstract

Although a yeast-based additive was initially employed as a performance enhancer, subsequent analysis revealed high aflatoxin B1 levels in the corn silage. Therefore, the objective of this study is to determine if the use of a yeast blend in the diet of Holstein calves that consumed feed naturally contaminated with high levels of aflatoxin can minimize the negative impacts of mycotoxins on animal health, contributing to improved performance. For this, we used 24 Holstein calves (6 months old) divided into two groups: Control (*n* = 12; no additive) and Treatment (*n* = 12; 5 g additive/animal/day). During the 100-day experiment, animals were weighed, feed intake was measured, blood samples were collected to assess health, and ruminal fluid was analyzed for ruminal fermentation. We observed greater weight gain and better feed efficiency in cattle that consumed the yeast-based additive compared to the control group. Yeast ingestion increased the concentration of propionic acid in the experimental environment, as well as increasing the protozoan count. Higher lymphocyte counts combined with higher levels of immunoglobulin G in the blood of females that consumed the additive were observed. Lower activity of enzymes that are biomarkers of liver damage, as well as markers of oxidative stress, was observed when animals consumed the yeast blend compared to the control group. Lower levels of ceruloplasmin (positive acute phase protein) and higher levels of transferrin (negative acute phase protein) are indicative of an anti-inflammatory response to the additive. The results preliminarily suggest that the consumption of the yeast blend is a nutritional tool capable of acting as a performance enhancer, even under challenging conditions, such as diets contaminated with aflatoxin at levels exceeding international limits.

## Simple Summary

Agriculture and livestock farming have faced challenges due to constant climate change, which has contributed to mycotoxin contamination in feed. Prolonged droughts or rainfall contribute to food with low nutritional quality, in addition to contamination by fungi, which, in grain or silage storage environments, tend to produce mycotoxins. In this study, aflatoxin contamination of corn silage caused negative impacts on the growth and liver health of calves. However, when a yeast blend was included in the diet, the negative impacts of the mycotoxin were avoided or minimized.

## 1. Introduction

The use of yeast-derived additives has established itself as an effective strategy in ruminant nutrition to promote intestinal health, modulate the immune response, and mitigate the adverse effects of feed contaminants, especially mycotoxins present in silage [1]. In terms of cattle nutrition, the combination of live yeasts with prebiotics (such as monosaccharides and β-glucans) has shown beneficial results related to intestinal health and stimulation of the immune system [1,2]. Autolyzed cytoplasmic fractions of yeasts can be available substrates for enterocytes and microorganisms of the gastrointestinal tract, which can accelerate intestinal mucosal renewal and favor microbial synthesis in the rumen [3]. Yeast cell walls deserve special attention due to their multifunctional properties, as they are mainly composed of β-glucans and mannan-oligosaccharides (MOS), components that act as immunomodulators and as physicochemical adsorbents of mycotoxins [4,5]. According to the literature, β-glucans interact with pattern recognition receptors (PRRs), such as Dectin 1 and certain Toll-like receptors, promoting the activation of macrophages, neutrophils, and NK cells, increasing phagocytosis and the production of regulatory cytokines, stimulating the innate immunity of cattle and reducing susceptibility to enteric infections [6]. In parallel, MOS exert an anti-adhesive action against pathogens that use mannose determinants for colonization, reducing the burden of enteric invaders and the occurrence of diarrheal conditions that compromise performance [7]. These actions improve animal health, but also increase the apparent digestibility of feed, raising feed efficiency, and reducing the variability of response to lower quality diets [1,8].

In the context of mycotoxins, it is known that the combination of autolyzed fractions and cell wall presents relevant synergistic effects, since the three-dimensional structure of the cell wall offers polar and hydrophobic sites capable of interacting with various mycotoxins, reducing their intestinal bioavailability and, consequently, systemic absorption that leads to immunosuppression, liver damage, reproductive disorders, and performance loss [9,10]. While the cell wall acts in the physical sequestration of toxins and in limiting their local action, the cytoplasmic fractions support mucosal recovery and provide substrates that aid in the restoration of digestive function and the metabolic support necessary to cope with toxic stresses [1]. Thus, reducing the load of bioavailable mycotoxins, coupled with strengthening innate defenses and improving intestinal integrity, tends to translate into less negative impact on weight gain, feed conversion, and reproductive parameters [11].

Beyond the direct effect on mycotoxins, the additive’s components indirectly influence the metabolic health of cattle, which was initially the main objective of our research. It is already known that additives can modulate the intestinal and ruminal microbiota could promote greater production of beneficial volatile fatty acids and optimize fiber degradation, increasing energy availability and favoring weight gain [12,13]. Furthermore, the attenuation of chronic inflammatory processes resulting from increased intestinal permeability or mycotoxin intoxication reduces the diversion of nutrients to costly immune responses, freeing up resources for growth and muscle deposition [14]. In practical systems, this combination of nutritional, immunological, and adsorbent effects reduces productive variability and offers resilience to forage contamination events, representing a nutritional management strategy with a positive economic impact [15].

Although the effects of aflatoxins have been extensively studied in adult dairy cows [16,17,18], knowledge regarding their impact on calves remains limited and not fully elucidated. Yeast and yeast-derived products, particularly Saccharomyces cerevisiae cell wall components, are widely recognized for their ability to adsorb aflatoxin B1 in the gastrointestinal tract, thereby reducing its bioavailability and toxic effects; however, the efficacy of this interaction is variable and not fully elucidated in vivo, especially in young ruminants. Therefore, since mycotoxins are a daily concern in animal feed, our hypothesis is that using a combination of yeasts and/or their fragments in the diet is an interesting strategy considering animal health and anti-mycotoxin effects. Thus, the objective of this study is to determine if the use of a yeast blend in the diet of Holstein calves that consumed feed naturally contaminated with high levels of aflatoxin can minimize the negative impacts of mycotoxins on animal health, contributing to improved performance.

## 2. Materials and Methods

We used a commercial product, Keypro Ultra^®^ (Tectron, Toledo, Brazil). The feed additive tested is based on dehydrated autolyzed sugarcane yeast, dehydrated autolyzed baker’s yeast, dehydrated autolyzed brewer’s yeast, and yeast cell wall. According to the manufacturer, it is a product with 340 g/kg of crude protein (min.), 10 g/kg of ether extract (min.), 100 g/kg of crude fiber (max.), 80 g/kg of mineral matter (max.), 99 to 5000 mg/kg of calcium, 6000 mg/kg of phosphorus (min.), 26 g/kg of lysine (min.), 6000 mg/kg of methionine (min.), 175 g/kg of beta-glucan (min.), 100 mg/kg of mannan-oligosaccharide (min.).

### 2.1. Facilities, Animals, and Diet

The study was conducted at the experimental farm of the State University of Santa Catarina, in the municipality of Guatambu, southern Brazil, under confinement conditions. The project was approved by the ethics committee on the use of animals in research at the State University of Santa Catarina (protocol number 8615191223), in accordance with the standards of the National Council for the Control of Animal Experimentation (CONCEA—Brazil).

Twenty-four Holstein calves, females, with an average age of 6 months old and weighing an average of 165 ± 6.14 kg, were used in this study. The animals were housed in collective pens (160 m^2^) of four calves per pen (a total of 6 pens: 3 control and 3 treatment), with concrete floors, and automatic waterers to provide water ad libitum. Each pen also had a feeder with dimensions that allowed the four animals in the pen to consume the feed without competing. This shed had a covered feeding area (1/5 of the pen), with the remainder outdoors. Lights were kept on at night in the feeding area; however, at no point during the experiment were the animals ventilated by fans.

The animals’ diet was based on concentrate and roughage (corn silage and chopped trifton hay) in a ratio of 42:58%, respectively. The diet was formulated for an average daily gain of 1 kg; the amount of food provided daily was weighed and mixed at two times of the day (8:00 am and 4:00 pm). As the diet was limited to a maximum weight gain of 1 kg per day, the feeding had a restrictive effect, causing the animals to consume everything that was provided (there was no leftover feed during the entire experiment).

### 2.2. Experimental Design

The animals were divided into two groups of 12 calves each, one control group receiving only the basal diet. The other group was defined as the “treatment,” with the yeast blend added to the concentrate. The distribution of animals within the group considered two variables: body weight and age, in order to have two homogeneous groups. These same criteria were used to divide the animals into pens, in order to avoid larger animals being housed together with smaller animals. The experimental period was 100 days, without an adaptation period because the animals were raised on the experimental farm and were already adapted to the diet of silage, concentrate, and hay used in our study.

Knowing the concentrate consumption of the animals, a mixture of the additive was used that allows for a dose of 5 g/animal/day, according to the manufacturer’s instructions. The additive was added to the concentrate during its production at the feed mill on the experimental farm. To ensure good mixing of the additive, a pre-mixture of the additive was made with 5 kg of ground corn in a Y-mixer; then the mixture was finalized in a horizontal mixer with a capacity of 300 kg.

Therefore, we used a completely randomized design (CRD) in this study. When evaluating performance variables (body weight, weight gain, feed intake, and feed efficiency), the pen was used as the experimental unit (3 per group); for hematological, biochemical, immunological, and ruminal fluid variables, the experimental unit was the animal (*n* = 12 per group).

### 2.3. Data and Sample Collection

Data and sample collection were carried out in the morning, before the first feeding of the day (no feed in the feeder for 14 h). There was no restriction on water for the animals during the night. All animals (*n* = 24; with *n* = 12 per group) were weighed individually, and a sample of blood and ruminal fluid was collected, as described below.

The animals were weighed on days 1, 63, 86, and 100 of the experiment using a digital scale. Knowing the body weight, the average daily weight gain (ADG) was calculated using a regression coefficient [19]. The daily feed intake (DFI) of each pen was monitored daily (feed provided—leftovers), with 100% of the feed provided daily being consumed, i.e., there was no leftover feed in the feeders throughout the entire experiment. Leftover food was assessed in the morning, before providing the first feeding of the day. This data allowed the calculation of feed efficiency: ADG/DFI.

Blood was collected on the same days as weighing, using vacuum collection tubes with anticoagulants of 4 mL (EDTA) for complete blood counts and without anticoagulant of 4 mL (Labtest, Lagoa Santa—MG, Brazil) to obtain the serum used in biochemical and immune analyses. The collected material was stored in a thermal box (12 °C) for transport to the laboratory. Upon arrival, within 3 h of collection, the complete blood count was performed, and the tube without anticoagulant was centrifuged (850× *g* for 10 min). The serum was collected and stored in microtubes in the freezer (−20 °C).

During weighing and blood collection, ruminal fluid was also collected. Using an esophageal probe, ruminal fluid was collected from calves on days 63 and 100 of the experiment. The pH of the collected sample was immediately measured using portable equipment. A volume of 20 mL was set aside for protozoan counting. The remaining material was filtered using cotton gauze; the material was stored in microtubes under freezing conditions (−20 °C).

### 2.4. Laboratory Analyses

#### 2.4.1. Feed Analyses and Measurement of Mycotoxins in Feed

During the experimental period, feed (silage, hay and concentrate) samples were collected in four days 1, 63, 86, 100 (same data collection date and samples) and kept frozen (−20 °C) until analysis. Before processing (day 7 after the experiment ended), the four samples were thawed and mixed separately by feed source (pool); these materials were used in the analyses described below. The samples were analyzed for dry matter (DM) after being in a forced-air circulation oven for 72 h at a temperature of 56 °C. We measured mineral matter (MM), crude protein (CP), and ether extract (EE) according to AOAC [20]. Neutral detergent fiber (NDF) and acid detergent fiber (ADF) concentrations were determined using the method of Van Soest et al. [21]. The chemical composition of the diets is shown in Table 1.

Mycotoxin levels (aflatoxins, fumonisins, zearelone, DON, T2, and ochratoxin) in silage and concentrate were measured using high-performance liquid chromatography with immunoaffinity purification, post-column derivatization, and fluorescence detection according to the methodology described by Müller et al. [22]. The methods showed a minimum limit of quantification (LOQ) of AFB1 and AFG1 = 2.0 µg/kg; DON = 70 µg/kg; FB1 = 70 µg/kg; ZEA = 20 µg/kg; OTA = 2 µg/kg; T-2 = 70 µg/kg. The level of natural contamination observed in corn silage was 22 µg of AFB1/kg of natural matter (6.9 µg of AFB1/kg dry matter); while in the concentrate, 274 µg of DON/kg and 89.2 µg of FB1/kg of dry matter were found. The other mycotoxins were not detected in these feeds.

#### 2.4.2. Hematological and Biochemical Analyses

Red blood cell count, total white blood cell count, hematocrit percentage, hemoglobin concentration, and leukocyte differentiation were performed immediately upon arrival at the laboratory, using an automated hematology analyzer (3 parts EQUIP VET 3000^®^, Equip, São Paulo, Brazil).

Serum levels of total protein (TP), fructosamine, albumin, cholesterol, triglycerides, and urea were analyzed, as well as the activity of the enzymes creatine kinase (CK-NAC), cholinesterase, alanine aminotransferase (ALT), gamma-glutamyltransferase (GGT), and aspartate aminotransferase (AST), using an automated analyzer (Zybio EXC 200^®^, Equip, São Paulo, Brazil) and commercial kits (Analisa^®^, Gold Analisa Diagnóstica S.A., Belo Horizonte, Brazil). Globulin levels were obtained using mathematical calculation (Globulin = total protein − albumin) [23].

#### 2.4.3. Oxidative Status

The levels of oxidative reactions were determined by the levels of thiobarbituric acid reactive substances (TBARS) and reactive oxygen species (ROS). Lipid peroxidation, measured by the amount of TBARS in serum, was determined using the method of Jentzsch et al. [24]. For the determination of reactive oxygen species (ROS), the technique described by Ali et al. [25] was applied. Antioxidant activity in blood was measured by glutathione S-transferase (GST) and glutathione peroxidase. Glutathione peroxidase (GPx) activity in serum was measured according to the methodology described by Paglia and Valentine [26]. All samples were analyzed in triplicate.

#### 2.4.4. Immunological Markers

Using a commercial kit (Labtest^®^, Labtest Diagnóstica S.A., Lagoa Santa, MG, Brazil), we measured the levels of immunoglobulins A and G (IgA and IgG) using the automated analyzer (Zybio^®^ EXC 200, Zybio Inc., Chongqing, China) and following the kit manufacturer’s instructions. All samples were analyzed in duplicate.

For protein fractionation, electrophoresis was performed on a polyacrylamide gel containing sodium dodecyl sulfate (SDS-PAGE), according to the technique suggested by Tomasi et al. [27] using a mini gel (10 × 10 cm). Using specific standards, we quantified the levels of ceruloplasmin, ferritin, and transferrin. All samples were analyzed in duplicate.

#### 2.4.5. Volatile Fatty Acid Profile in the Rumen

Ruminal fluid samples were thawed at 5 °C and manually shaken for homogenization. Aliquots of 1 mL of the supernatant of ruminal fluid samples were collected in polypropylene microtubes (2 mL) and then centrifuged for 5 min (12,300× *g*). Then, 250 μL of the supernatant was transferred to a new microtube containing 250 μL of formic acid. The mixture was manually shaken and centrifuged for 3 min. After centrifugation, 250 μL of the supernatant of the mixture were collected in another polypropylene tube previously containing 500 μL of isoamyl alcohol solution (692.40 μg mL^−1^ in methanol), used as an internal standard, and were homogenized and centrifuged again. 650 μL of sample was inserted into a 2 mL injection vial. 1 μL of extract was injected into a gas chromatograph equipped with a flame ionization detector (GC-FID; Varian Star 3400, Palo Alto, CA, USA) and an autosampler (Varian 8200CX, Palo Alto, CA, USA) in split mode (1:10) at 250 °C. The carrier gas used was hydrogen at a constant pressure of 10 psi. The analytes (acetic, propionic, butyric, valeric and isovaleric acids) were separated by a CP-Wax 52CB capillary column (50 m × 0.32 mm; 0.20 μm stationary phase thickness). The initial column temperature was set at 40 °C for 1 min and increased to 100 °C at a rate of 10 °C min^−1^, then to 110 °C for 3.5 °C min^−1^, and, for last, to 230 °C at a rate of 20 °C min^−1^, where it remained for 1 min. The detector temperature was set to 250 °C. Method validation comprised the following parameters: selectivity, linearity, linear range, repeatability, precision, limit of detection (LOD) and limit of quantification (LOQ) for acetic, propionic, butyric, and isovaleric acids. Linearity was assessed by calculating a regression equation using the method of least squares. LOD and LOQ values were obtained by sequential dilutions up to signal-to-noise ratios of 3:1 and 6:1, respectively. Precision was assessed by analyzing the repeatability of six replicated samples. Accuracy was determined by recovering known amounts of the standard substances added to a diluted sample (Table S1). Valeric acid was expressed as equivalent of isovaleric acid. The results were expressed in mol 100 mol^−1^ of each SCFA in the ruminal fluid.

#### 2.4.6. pH and Protozoan Count in Ruminal Fluid

To measure the pH, the ruminal fluid was immediately placed in a container (universal collector) without filtration, where the measurement was performed with a portable digital pH meter (Testo 205^®,^ Testo SE & Co., KGaA, Lenzkirch, Germany). A 10 mL aliquot of the unfiltered ruminal fluid was used to quantify the protozoa. After placing the sample in a 50 mL falcon tube, we added 10 mL of 50% formalin to the sample and then removed 1 mL of this and placed it in a 15 mL falcon tube. Then, two drops of brilliant green were added, and the product was left to stand for at least 4 h. After staining, 9 mL of 30% glycerol was added. The reading was performed using 1 mL in a Sedgewick-Rafter counting chamber, according to the methodology of Dehority [28], with a binocular biological microscope.

### 2.5. Statistical Analysis

The data showed a normal distribution after being subjected to the Shapiro–Wilk test; in addition, skewness, kurtosis, and homogeneity were evaluated using Levene’s test; linearity was assessed using linear regression. Based on these preliminary results, our data were analyzed using the SAS MIXED procedure (SAS Inst. Inc., Cary, NC, USA; version 9.4), with covariance structure compound symmetry to determine the denominator degrees of freedom for the fixed effects test (day, treatment, and treatment × day interaction) in a completely randomized design to determine ADG and feed efficiency. Body weight and feed intake were analyzed as repeated measures and tested for fixed effects of treatment, day, and treatment × day interaction (group) and pen as random variables. All other variables (serum biochemistry, blood count, and ruminal fluid) were analyzed as repeated measures and tested for fixed effects of treatment, day, and treatment × day interaction (group) and animal as random variables. All results obtained on day 1 (d1) for each variable were also included as covariates, as was initial body weight; however, the covariate command was removed from the model when *p* > 0.05. Means were separated using the PDIFF method (*t*-test), and all results were reported as LSMEANS followed by the standard error of the mean (SEM). All results were presented as mean considering significance when *p* ≤ 0.05.

## 3. Results

### 3.1. Performance

In this study, the addition of a blend based on autolyzed yeast and cell wall enhanced the final body weight at 100 days and the average daily gain (ADG) of the animals (Table 2). However, there was no difference between the groups regarding feed consumption, showing that the observed effects resulted from greater efficiency in nutrient utilization and not from increased intake. Feed efficiency was higher in the treated group, demonstrated by the lower feed consumed/weight gain ratio, which confirms better conversion of nutrients into body mass in the animals that consumed the additive.

### 3.2. Complete Blood Count

Lymphocyte count was higher in animals that consumed the yeast blend (treatment effect; *p* < 0.05—Table 3), with no effect of day or treatment × day interaction for this variable. The other complete blood count variables did not differ between the groups (*p* > 0.05), information regarding the number of erythrocytes, hemoglobin concentration, hematocrit percentage, MCV, MCHC, leukocyte count, monocytes and granulocytes (Table 3).

### 3.3. Serum and Oxidative Biochemistry

**Figure 1:**
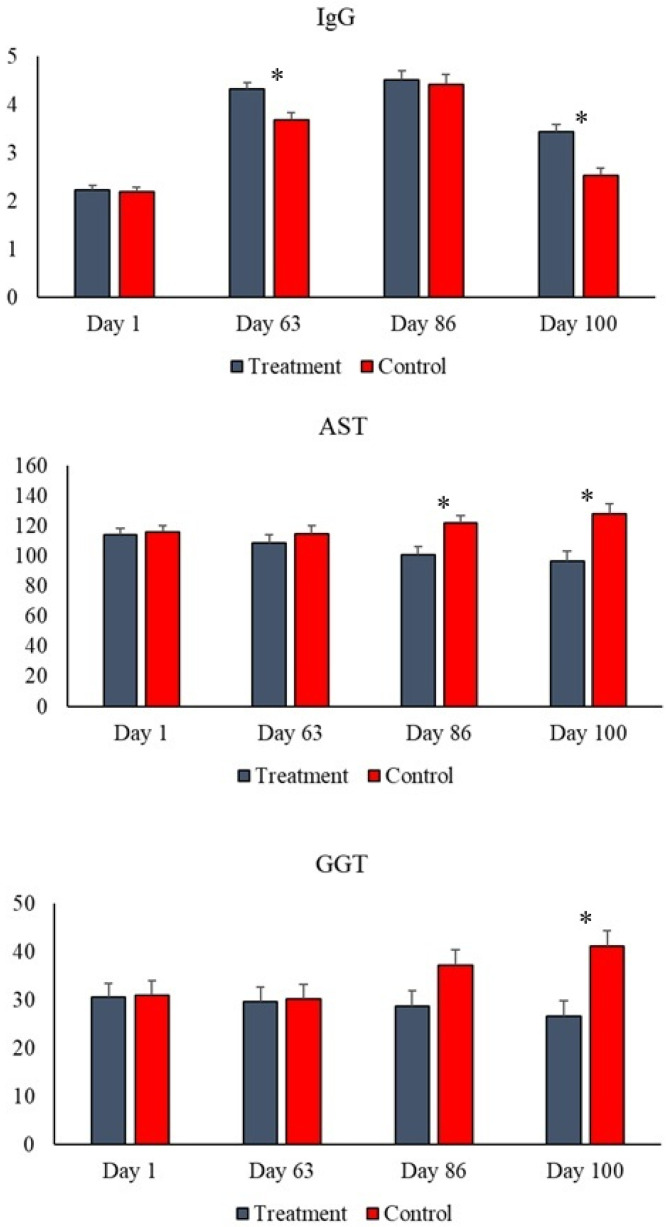
Treatment × day interaction for immunoglobulin G (IgG—g/dL) levels and activity of aspartate aminotransferase (AST—U/L) and gamma-glutamyltransferase (GGT—U/L) enzymes. Difference between groups (*p* < 0.05) on the day was illustrated by the presence of an asterisk (*).

Clinical and metabolic biochemistry results are presented in Table 4. No treatment effect, day effect, or treatment × day interaction was observed for the variables albumin, globulins, total protein, creatine kinase, urea, cholesterol, triglycerides, cholinesterase, and fructosamine. There was a treatment effect on ALT and AST, both with lower activities in the treatment group compared to the control. A day effect and treatment × day interaction were also observed for AST and GGT on days 86 and 100 (Figure 1), with the activity of these two enzymes being lower in the serum of animals that consumed the additive.

**Figure 2:**
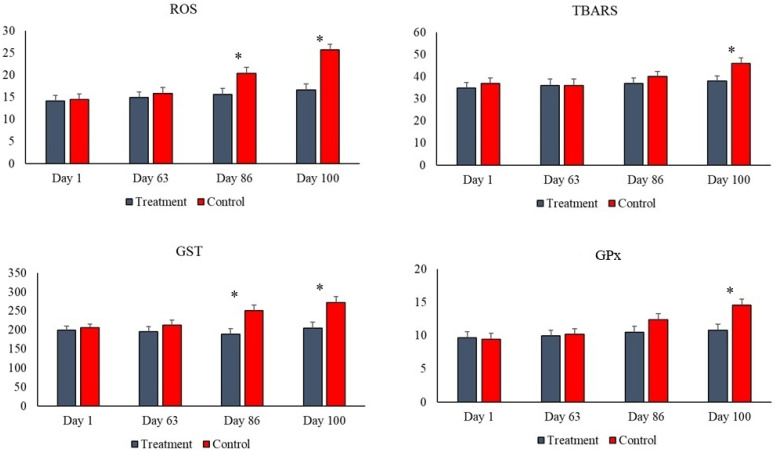
Treatment × day interaction for levels of reactive oxygen species (ROS—U fluorescence) and lipid peroxidation (TBARS—nmol/mL), as well as in the activity of the antioxidant enzymes glutathione S-transferase (GST—μmol Cdnb/min) and glutathione peroxidase (GPx—U/mg protein). Difference between groups (*p* < 0.05) on the day was illustrated by the presence of an asterisk (*).

Oxidative biochemistry results are presented in Table 4 and Figure 2. TBARS levels and GPx activity showed a day effect and treatment × day interaction (d100), being lower in the serum of animals that consumed the additive compared to the control. GST activity and ROS levels were lower in the treatment group compared to the control group, with a treatment and day effect being observed, as well as a treatment × day interaction (d86 and 100).

### 3.4. Immunological Markers

Immunological markers had results shown in Table 4. No treatment, day, or treatment × day interaction effect was observed for the variables IgA, and ferritin (*p* > 0.05). However, higher levels of IgG were observed in the serum of animals that consumed the yeast blend on days 63 and 100 compared to the control group (Figure 1). The treatment effect for ceruloplasmin showed lower levels of this acute-phase protein in the serum of the treatment animals; the concentration of transferrin was higher in these same animals compared to the control group (Table 4).

### 3.5. pH, Protozoa, and Volatile Fatty Acid Profile in Rumen Fluid

Rumen fluid results are shown in Table 5. There was no treatment effect on pH (*p* > 0.05). Protozoa counts showed a day effect in the control group, decreasing over the days. In the treatment × day interaction, a higher number of protozoa was observed in the rumen of animals that consumed the additive compared to the control group. No treatment or day effect was observed for any of the volatile fatty acids, but there was a treatment × day interaction for propionic acid, isovaleric acid, and total VFAs on day 63. Isovaleric acid was lower in the treatment group, while the concentration of propionic acid was higher in the rumen of animals that consumed the additive. In addition, a higher concentration of VFAs was observed in the rumen fluid of animals in the treatment group compared to the control group on day 63.

## 4. Discussion

The contamination of corn silage with 22 µg/kg of AFB1 was concerning, and it should be noted that it was not planned for this experiment. Although this quantity is below the Brazilian limit for ruminants (MAPA—IN 113/2020—up to 50 µg/kg), it is above the limits of the European Union (Directive 2002/32/EC—up to 5 µg/kg), the United States (FDA—up to 20 µg/kg at 2019), and the Codex Alimentarius (FAO/WHO—up to 20 µg/kg). A recent review highlighted the influence of climate change on aflatoxin contamination in feed, which can generate global food insecurity; because, according to researchers, fungi such as Aspergillus flavus produce aflatoxins, which can seriously harm health, compromising the immune system and causing chronic diseases, and how temperature and humidity affect the production of this mycotoxin [29]. These favorable conditions for mycotoxins are common in southern Brazil, where the experiment took place, occurring in corn silage from a plant that underwent a 40-day drought period in the final production phase, which affected the silage quality as can be seen in Table 1. In our study, it is evident that the presence of AFB1 in the diet affected weight gain, since the diet was formulated for an ADG of 1 kg, with animals in the control group having lower gain (0.92 kg), while the animals that consumed the yeast blend managed to have a weight gain (1.06 kg) higher than estimated in the formulation.

Literature indicates that yeast cytoplasmic fractions provide nucleotides, peptides, and B vitamins that promote intestinal mucosal renewal and energy metabolism, enhancing nutrient absorption [1], which may explain the improved animal performance when consuming the additive. Furthermore, β-glucans and mannan-oligosaccharides present in the yeast cell wall exert an immunomodulatory action, contributing to the reduction of intestinal inflammatory processes and the maintenance of hepatic and digestive function, and may also act as adsorbents of mycotoxins [6,30,31]. Therefore, all these mechanisms minimized the negative effects of AFB1, consequently leading to greater weight gain and feed efficiency.

The stability of ruminal pH in both groups indicates maintenance of acid-base homeostasis with the inclusion of the yeast blend. However, on day 100 the protozoan count was higher, while the sum of volatile fatty acids was higher on day 63, due to an increase in propionate; and despite the reduction in isovaleric acid. Microecology studies have shown that protozoa strongly influence VFA production and that their interactions with the bacterial community depend on the identity and size of the species [32,33]. Thus, changes induced by the supplement, by alteration of the ruminal redox potential, by the supply of specific substrates or by the adsorption of toxic compounds, can cause functional substitutions in the microbiota that are temporarily reflected in the fermentative profile. These fermentative changes can, in turn, mediate systemic effects observed later, such as the recovery of redox balance and liver improvement [33,34]. According to the literature, AFB1 represents a serious threat to livestock health, despite ruminants having ruminal microorganisms capable of degrading AFB1 [35]. However, it is known that this capacity is limited, especially when the amount of mycotoxin is high in the diet; under these conditions, it negatively affects microbial protein synthesis in the rumen, resulting in a negative protein balance [36]. We cannot rule out the effect of AFB1 on the ruminal environment, but our main hypothesis is an effect of the yeasts consumed via additive, since a meta-analysis of 110 articles showed that yeast intake increases the volatile fatty acid profile [37], as observed in our research.

The findings demonstrate a specific lymphocytic response associated with yeast consumption via commercial product, without alterations in the erythrocyte profile. This immunomodulatory action is already well known in yeast ingestion [1,6,38]. Experiments with yeast derivatives and nucleotides report support for the proliferation and viability of peripheral blood mononuclear cells (PBMCs) and greater functional competence of mononuclear cells, suggesting that yeast ingestion stimulates adaptive and cellular competence without inducing systemic inflammation [39]. This immunological profile is compatible with the late increase in IgG observed in this study, indicating sustained stimulation of the humoral response and possible improvement in immunological memory [40].

Modulation of seric proteins was observed, with lower levels of ceruloplasmin (positive acute phase protein) and higher levels of transferrin (negative acute phase protein) in the treatment group, characterizing, along with the other variables, an anti-inflammatory effect, due to lower basal inflammatory activation and improved iron transport capacity. Together with the increase in IgG, these signals point to an effective reallocation of the body’s metabolic resources: less energy spent on inflammatory processes and more available for growth and adaptive defense [31,39].

Although many metabolic variables such as albumin, globulins, creatine kinase, cholesterol, cholinesterases, and urea were not altered by the additive treatment, nor by the ingestion of silage with aflatoxin, as the results remained within normal limits [23]. However, when the animals consumed the yeast blend, lower serum activity of the enzymes ALT, AST, and GGT was observed compared to the control, enzymes that indicate liver damage and liver function, considered a hepatoprotective effect of the additive against the aflatoxin ingested in the corn silage. This effect is directly associated with lower levels of ROS and TBARS also in the serum, since there is a strong relationship between oxidative reactions and inflammation, and therefore both are at lower levels in the animals that consumed the yeast via the additive. Confirming this hypothesis, the activity of two glutathione enzymes with antioxidant power (GPx and GST) is lower in the blood of these animals in the treatment group, which is understandable and consistent with the other results regarding liver health and oxidative stress. These findings converge with studies showing increased plasma antioxidant capacity and greater activity of SOD and GSH-Px after animals consume yeast and microalgae, an effect that, according to the authors, can be potentiated by the addition of nucleotides and the presence of organic selenium in commercial formulations [39,41]. According to the literature, mechanisms of adsorption and reduction of mycotoxin bioavailability by the yeast cell wall, decreased endotoxin translocation due to improved intestinal mucosal integrity, and enhanced antioxidant defenses through the action of microalgae and selenium result in less liver damage and reduced generation of free radicals [5,42,43].

Classic studies demonstrate that the yeast cell wall has the capacity to adsorb mycotoxins, reducing their intestinal bioavailability, and recent studies indicate that blends with antioxidant and probiotic action amplify this protection through microbiota modulation and reinforcement of antioxidant defenses [9,44]. This convergence between experimental evidence and published findings lends robustness to the argument that the tested intervention is effective in mitigating the effects of mycotoxins. We were unable to define which of these mechanisms was involved or whether both were contributing to minimizing the negative impacts of AFB1; however, what is important at this moment is to know that a yeast blend as an additive is an important nutritional tool today, due to climate impacts contributing to increased contamination of animal feed by mycotoxins.

Following the conclusion of the formal experiment, an incidental observation was made regarding fertility of animals, i.e., 3 months after the end of the experiment. At 13 months of age, the animals underwent a fixed-time artificial insemination protocol, with all animals inseminated with semen from the same bull, from the same batch. The protocol was the same for all heifers, but between 19 and 21 days, a large number of heifers were found to be in estrus; these animals were then subjected to natural mating. Thirty days after insemination, a pregnancy diagnosis was made via ultrasound, where 7/12 heifers in the treatment group were pregnant, as were 2/12 heifers in the control group (one of these heifers in the control group aborted at 100 days of gestation). The pregnancy rate for heifers in both groups was low, but that of the control group was excessively low (16.6%). This information is from after the end of the experiment; however, I consider it important to include this data in the discussion of this work; however, it is important to make it clear that this is preliminary information and needs to be investigated in future studies. A study published in 2021 mentions that constant ingestion of aflatoxin affects sperm, oocytes, and pre-implantation embryonic development, suggesting possible embryonic loss in cows [17]. This information is consistent with data from our research, where the ingestion of silage with aflatoxin interfered with fertility; but we still need to be cautious about this hypothesis.

## 5. Conclusions

The inclusion of the yeast blend was able to mitigate the negative impacts associated with high levels of aflatoxin in the diet, as these animals showed improved performance in terms of weight gain and feed efficiency. Furthermore, animals that consumed the yeast had better results in biochemical parameters related to health, i.e., lower activity of liver enzymes (AST, ALT, and GGT) and levels of oxidative stress markers (ROS, TBARS, GST, and GPx), which can be interpreted as benefits of the additive as discussed. Another benefit of the additive is related to a greater immune response (IgG) while the inflammatory process was lower, which may also explain the greater weight gain, since animals with less inflammation consequently expend less ATP in this inflammatory response; ATP that is used to improve performance. Finally, we conclude that the consumption of the yeast additive alters the rumen environment, notably resulting in higher counts of protozoa and propionic acid.

## Figures and Tables

**Table 1:** Ingredients and composition of the diet provided to Holstein heifers during rearing.

Variables	Corn Silage	Hay	Concentrate ^1^
Dry matter—DM (%)	31.40	85.30	88.40
Ash (% DM)	4.98	6.31	8.65
Ether extract (% DM)	2.21	0.86	3.37
Crude protein (% DM)	4.10	2.97	17.10
NDF (% DM)	62.80	76.20	25.90
ADF (% DM)	37.10	31.00	9.76
Starch (% DM)	21.30	-	31.10

Note ^1^: The concentrate used in the diet was formulated based on ground corn (41.3%), soybean meal (14.1%), wheat bran (23.3%), soybean hulls (14.4%), mineral and vitamin supplement—Bovigold (4.09%) and common salt (2.61%).

**Table 2:** Performance of Holstein heifers fed a yeast blend.

Variables	Control (*n* = 3)	Treatment (*n* = 3)	SEM	*p*-Value ^1^
Body weight (kg)				
Initial	163	168	6.12	0.85
Final	256 ^b^	274 ^a^	7.31	0.05
ADG, kg	0.92 ^b^	1.06 ^a^	0.02	0.03
DMI, kg MS	5.89	5.79	0.16	0.98
Feed efficiency, kg/kg	0.156 ^b^	0.183 ^a^	0.01	0.02

Note ^1^: treatment effect, with the difference illustrated by different letters (^a,b^) on the same line (*p* ≤ 0.05).

**Table 3:** Blood count of Holstein heifers fed a yeast blend.

Variables	Control (*n* = 12)	Treatment (*n* = 12)	SEM	*p*-Treat ^1^	*p*-Day ^2^	*p*-Treat × Day ^3^
White blood cells (×10^3^ µL)	13.40	14.20	1.46	0.87	0.64	0.76
Lymphocytes (×10^3^ µL)	7.68 ^b^	9.01 ^a^	1.14	0.05	0.17	0.06
Granulocytes (×10^3^ µL)	4.55	3.98	0.33	0.46	0.27	0.52
Monocytes (×10^3^ µL)	1.21	1.25	0.12	0.93	0.81	0.89
Red blood cells (×10^6^ µL)	7.33	7.28	0.12	0.97	0.93	0.98
Hemoglobin (g/dL)	10.90	10.9	0.17	0.98	0.95	0.98
Hematocrit (%)	30.60	30.8	0.39	0.97	0.96	0.97
Platelets (×10^3^ µL)	349	330	31.7	0.86	0.75	0.81

Note ^1^: Treatment effect, with the difference illustrated by different letters (^a,b^) on the same line (*p* ≤ 0.05). Note ^2^: There was no effect of the day (*p* > 0.05); Note ^3^: There was no treatment × day interaction (*p* > 0.05).

**Table 4:** Serum biochemistry, oxidative status, and immunological markers in Holstein heifers fed a yeast blend.

Variables	Control (*n* = 12)	Treatment (*n* = 12)	SEM	*p*-Treat ^1^	*p*-Day ^2^	*p*-Treat × Day ^2^
Albumin (g/dL)	3.04	2.97	0.05	0.92	0.74	0.82
Globulin (g/dL)	2.94	2.92	0.07	0.94	0.86	0.89
Total protein (g/dL)	5.98	5.90	0.09	0.96	0.91	0.93
Creatine kinase (U/L)	255	274	29.10	0.85	0.61	0.52
Cholinesterase (U/L)	2514	2462	88.10	0.56	0.34	0.29
Cholesterol (mg/dL)	71.10	74.90	2.36	0.77	0.83	0.81
Fructosamine	303	298	5.79	0.89	0.92	0.92
Urea (mg/dL)	35.40	34.30	0.94	0.95	0.96	0.95
IgA (mg/dL)	9.99	9.88	0.31	0.87	0.81	0.84
IgG (g/dL)	3.88	4.08	0.18	0.38	0.04	0.05
Ceruloplasmin (g/dL)	0.83 ^a^	0.67 ^b^	0.02	0.05	0.11	0.26
Ferritin (g/dL)	0.21	0.22	0.01	0.94	0.93	0.96
Transferrin (g/dL)	0.25 ^b^	0.36 ^a^	0.01	0.03	0.07	0.11
AST (U/L)	125 ^a^	102 ^b^	5.41	0.05	0.01	0.01
ALT (U/L)	12.40 ^a^	8.98 ^b^	0.84	0.05	0.13	0.17
GGT (U/L)	39.40	28.70	3.25	0.09	0.01	0.01
ROS (U fluorescence)	21.50 ^a^	15.60 ^b^	1.35	0.02	0.01	0.01
TBARS (nmol/mL)	42.60	37.10	2.45	0.34	0.05	0.02
GST (μmol Cdnb/min)	254 ^a^	195 ^b^	14.10	0.01	0.01	0.01
GPx (U/mg protein)	12.40	10.10	0.96	0.42	0.01	0.01

Note ^1^: Treatment effect, when the difference between groups was illustrated by different letters (^a,b^) on the same line (*p* ≤ 0.05). Note ^2^: The day effects and treatment × day interaction, when significant (*p* < 0.05), the results are presented in Figure 1 and Figure 2.

**Table 5:** pH, number of protozoa and volatile fatty acid (VFA) profile in the ruminal fluid of Holstein heifers fed a yeast blend.

Variables	Control (*n* = 12)	Treatment (*n* = 12)	SEM	*p*-Treat ^1^	*p*-Day ^3^	*p*-Treat × Day ^2^
pH				0.95	0.96	0.95
d63	6.63	6.64	0.03			
d100	6.64	6.66	0.04			
Protozoan (×10^7^ n°/L)				0.75	0.01	0.01
d63	64.1	62.5	3.36			
d100	36.1 ^b^	51.7 ^a^	3.67			
Acetic acid (mmol/L)				0.96	0.98	0.93
d63	63.2	63.1	0.36			
d100	65.1	64.3	0.37			
Propionic acid (mmol/L)				0.89	0.79	0.03
d63	9.6 ^b^	12.81 ^a^	0.24			
d100	10.5	12.3	0.31			
Butyric acid (mmol/L)				0.91	0.72	0.94
d63	8.66	8.76	0.29			
d100	9.02	9.1	0.3			
Isovaleric acid (mmol/L)				0.68	0.19	0.05
d63	1.71 ^a^	1.52 ^b^	0.04			
d100	1.64	1.62	0.03			
Valeric acid (mmol/L)				0.86	0.73	0.89
d63	1.16	1.03	0.03			
d100	1.11	1.14	0.03			
Total VFA (mmol/L)				0.86	0.81	0.05
d63	84.3 ^b^	87.2 ^a^	0.39			
d100	87.4	88.5	0.45			

Note ^1^: There was no treatment effect on these variables (*p* > 0.05). Note ^2^: Treatment × day interaction was illustrated by different letters (^a,b^) on the same line on the day when there was a difference between groups (*p* < 0.05). Note ^3^: Day effect only on protozoan count and only in the control group.

## Data Availability

The data are with the authors and can be available upon request.

## Supplementary Materials

The following supporting information can be downloaded at: https://www.mdpi.com/article/10.3390/ani16020219/s1, Table S1. Standardization of methodology for analyzing the profile of short-chain fatty acids in the ruminal fluid of Holstein calves during rearing.
