# Supplementary material for: Adding a Yeast Blend to the Diet of Holstein Females Minimizes the Negative Impacts of Ingesting Feed Naturally Contaminated with Aflatoxin B1
Source: Animals (Basel). 2026 Jan 12;16(2):219. doi: 10.3390/ani16020219 (PMC12837908; doi:10.3390/ani16020219)
Supplement: Supplementary file 1 [file animals-16-00219-s001.zip › animals-4045235-supplementary.pdf]

Table S1. Standardization of methodology for analyzing the profile of short-chain fatty acids in the ruminal fluid of Holstein calves during rearing.

	Acetic acid	Propionic acid	Butyric acid	Isovaleric acid
R^2	0.9995	0.9995	0.9998	0.9999
Equation	y = 0.0094x + 0.0057	y = 0.0163x - 0.0228	y = 0.0241x - 0.0081	y = 0.0308x - 0.0061
Linear range (mmol L^{-1})*	5.08 - 101.62	1.83 - 73.34	1.45 - 46.38	0.70 - 22.30
LOD (mmol L^{-1})	1.27	0.92	0.72	0.35
LOQ (mmol L^{-1})	2.54	1.83	1.45	0.70
Accuracy	105.96	109.16	104.11	103.39
Repeatability (RSD)	2.76	4.30	1.66	2.54

* The linear range. LOD (limit of detection) and LOQ (limit of quantitation) were expressed in mmol of SFA for L of ruminal fluid.
